# What the Inbred Scandinavian Wolf Population Tells Us about the Nature of Conservation

**DOI:** 10.1371/journal.pone.0067218

**Published:** 2013-06-21

**Authors:** Jannikke Räikkönen, John A. Vucetich, Leah M. Vucetich, Rolf O. Peterson, Michael P. Nelson

**Affiliations:** 1 Swedish Museum of Natural History, Department of Contaminant Research, Stockholm, Sweden; 2 School of Forest Resources and Environmental Science, Michigan Technological University, Houghton, Michigan, United States of America; 3 Department of Forest Ecosystems and Society, Oregon State University, Corvallis, Oregon, United States of America; Lund University, Sweden

## Abstract

The genetic aspects of population health are critical, but frequently difficult to assess. Of concern has been the genetic constitution of Scandinavian wolves (*Canis lupus*), which represent an important case in conservation. We examined the incidence of different congenital anomalies for 171 Scandinavian wolves, including the immigrant founder female, born during a 32-year period between 1978 and 2010. The incidence of anomalies rose from 13% to 40% throughout the 32-year study period. Our ability to detect this increase was likely facilitated by having considered multiple kinds of anomaly. Many of the found anomalies are likely associated with inbreeding or some form of genetic deterioration. These observations have implications for understanding the conservation needs of Scandinavian wolves. Moreover, these observations and the history of managing Scandinavian wolves focus attention on a broader question, whether conservation is merely about avoiding extinction of remnant populations, or whether conservation also entails maintaining genetic aspects of population health.

## Introduction

The restoration and maintenance of population health is a central goal of conservation. Population health is widely considered to entail rates of survival and reproduction and a mean abundance level that all correspond to a high probability of persistence. It is also widely appreciated that survival and reproduction can be reduced by genetic deterioration [1], and that effective conservation requires populations to be sufficiently large to avoid these risks [2].

While these principles are generally endorsed, what counts as appropriate application of these principles varies greatly among conservation professionals. For example, a significant body of conservation literature suggests conservation targets routinely fall short of numbers necessary to maintain the genetic aspects of population health necessary for long-term persistence [2]. Some conservation professionals seem to believe, however, that genetic concerns arise from theoretical and laboratory results, and are not generally applicable to real populations of conservation concern [3], [4]. Other conservation professionals respond by invoking the precautionary principle, arguing that if genetic aspects of health are protected only after their relevance is demonstrated in each particular case, then populations can incur irreversible damage [5].

Many recovery plans do not apply the precautionary principle, favoring instead conservation goals that are palatable to stakeholders who may not even support conservation [6]. Consequently, there are concerns that political interests too frequently override what the best available science suggests is appropriate conservation (e.g., [7]).

These observations suggest the meaning of population health and what counts as evidence for population health are contested within the conservation community. As such, the meaning of conservation is contested insomuch as conservation is about the maintenance and restoration of population health [8], [9]. Indetermination about the meaning of conservation and failure of many outside the conservation community to value conservation combine to form a crippling obstacle for successful conservation.

These circumstances are especially troublesome for large carnivore conservation. The widespread loss of large carnivores and their ecosystem-wide effects may well represent humankind's most pervasive influence on nature [10]. Carnivores are difficult to conserve because the existence of carnivores creates conflict with humans over the consequences (real and imagined) of their existence [11], [12].

Here, we present new evidence showing how a carnivore population of prominent conservation concern, the inbred Scandinavian wolf (*Canis lupus*) population, is exhibiting an increasing trend of anomalies. These observations have implications for better understanding what counts as population health, what counts as evidence of population health, and by extension what counts as conservation.

### The study population

Wolves were once widespread throughout Scandinavia. After decades of persecution by humans, the Scandinavian wolf population had become functionally extinct by the mid-1960s when the last known reproduction had taken place [13]. Wolves were protected in Norway and Sweden shortly afterward. Wolves recolonized southern Scandinavia in the early-1980s, when a pair of wolves immigrated from the Finnish-Russian population [13], [14].

Genetic aspects of population health have been of chronic concern for the Scandinavian wolf population because the population was founded from just a few individuals and is largely isolated from the Finnish-Russian population [14].

Given the population's census size in recent years (between 289 and 325, [15]), and given recent estimates that the populations' genetically effective size (N_e_) is approximately 0.2 times the census size [16], the N_e_ of Scandinavian wolves has only recently grown to about 80. Consequently, the Scandinavian population has significantly less genetic variability than wolves from Finland [14] and a rate of inbreeding that is equivalent to offspring from a full sibling mating (i.e., *F* = 0.27; [17]). Juvenile survival is lower among Scandinavian wolves with higher rates of inbreeding [18] and an elevated incidence of congenitally malformed vertebrae in comparison with the non-inbred Finnish/Russian wolves have been reported in the population [19].

Effective immigration into the population, which could mitigate genetic deterioration, has been rare, occurring on just three occasions in the past three decades [14], [20]. Wolf-human conflicts have been a concern for the conservation of Scandinavian wolves. Immigration is limited by high rates of human-caused mortality in northern Sweden and Finland where wolves are in conflict with humans herding reindeer, *Rangifer tarandus* [21]. Human-assisted immigration from other countries has been suggested, but not yet implemented, to substitute for natural immigration [22]. Only translocation of immigrant wolves from conflict areas in northern Sweden has been carried out. One female has been translocated unsuccessfully four times to southern Sweden and is now (April 2013) once again in her old territory [23]. Also a young pair of immigrants has been translocated by car about 745miles (1200 km) from the northern areas [24]. However, it remains uncertain whether these individuals will become genetically effective immigrants. The population's recovery has also been significantly slowed by the impact of poaching on population growth [25].

Despite the effects of illegal poaching and genetic deterioration, wolf abundance generally increased from 1980 to 2010 [20]. By 2009, abundance had grown to 210 wolves, which had been a temporary conservation goal of the Swedish government until 2012. In 2010, the Swedish government began managing a harvest of wolves designed to prevent abundance from increasing beyond about 210 wolves. This decision was strongly influenced by the purported value of gaining social tolerance from livestock owners and other groups [26].

## Materials and Methods

We examined the relationship between year of birth and incidence of congenital pathology for 171 Scandinavian wolves born during a 32-year period between 1978 and 2010. The ages of the specimens were based on counting annual cementum annuli in canine teeth (conducted by Matson's cementum age analysis Laboratory, Montana, USA) or from museum specimen data. The age determination should be seen as an approximate since accuracy errors may sometimes occur in age analysis [27].

We also characterized each wolf according to the presence or absence of various kinds of congenital anomalies. We examined osteological material and characterized congenital vertebral anomalies in 122 wolves and included an additional 49 wolves analyzed in [19]. Each vertebral segment of the vertebral columns was individually analyzed (see also [19], [28]).

Of the wolves in our sample, 131 included sufficient materials to also assess dental anomalies. We examined each of these 131 samples for malocclusions (deviations from the normal scissor bite) including: abnormal number of teeth (supernumerary teeth and hypodonty, i.e. missing teeth), malposition of teeth, and other abnormal patterns.

Finally, we also characterized each sample according to the presence or absence of congenital heart defects, cryptorchidism (a testicular defect) and kidney malformations. These conditions were reported in necropsy reports published by the Swedish National Veterinary Institute.

No new samples were collected and no wolves were sacrificed for this study. All specimens were provided by the Swedish Museum of Natural History and were used with permission. The wolf is a protected species in Sweden and belongs to the “state's wildlife,” which means that they must be turned over to the state if they are found dead or killed. According to law, all dead wolves in Sweden go through necropsy at the National Veterinary Institute, SVA. After this, the wolves are sent to the Swedish Museum of Natural History, which provides repository for all wolf specimens.

## Results

### Necropsy results

Of the 171 necropsied wolves, twenty-nine (17.0%) exhibited some kind of vertebral anomaly, which included 4 with more than one anomaly (Table 1). The most prevalent congenital vertebral anomaly was lumbosacral transitional vertebrae, LSTV (Table 1). This is an anomaly that can be benign, found as a clinically incidental finding [29] or that can lead to clinical problems [30]–[32].

**Table 1:** Vertebral and dental anomalies found in Scandinavian wolves born between 1978 and 2010.

Category (sample size)	Type of Anomaly	Frequency (sample size)
Vertebral Anomalies (171)	Anomaly of the 6^th^ cervical vertebra	2.3% (4)
	Blockvertebra	0.6% (1)
	Cervical transitional vertebra (the 7^th^)	1.8% (3)
	Hemivertebra	0.6% (1)
	Lumbosacral transitional vertebra	8.8% (15)
	Sacrococcygeal transitional vertebra	2.3% (4)
	Reduction of a lumbar vertebra (from 7 to 6)	2.3% (4)
	Thoracolumbar transitional vertebra	1.8% (3)
Dental Anomalies (131)	Hypodonty	6.1% (8)
	Malformed tooth	0.8% (1)
	Mesioversion of a canine	2.3% (3)
	Microdontia	0.8% (1)
	Persistent primary teeth	0.8% (1)
	Rotated teeth	4.6% (6)
	Supernumerary teeth	3.1% (4)

Of the 131 wolves whose dental characteristics were investigated, twenty-one (16.0%) exhibited some kind of dental anomaly that included 3 with more than one anomaly (Table 1). Eighteen (13.7%) of 131 individuals exhibited some kind of dental malocclusion:

- Levelbite (n = 2), when upper and lower incisors meet edge on edge, which can cause severe attrition of the teeth.
- Anterior crossbite (n = 1), when one (or more) mandibular incisors is positioned labial to the maxillary incisors.
- Traumatic tooth-to-tooth malocclusions (n = 11), a mild anomaly, which resulted in moderate to severe attrition of the canines.
- Overshot (n = 4), when the maxilla is too long or the mandibula is too short.

**Figure 1:**
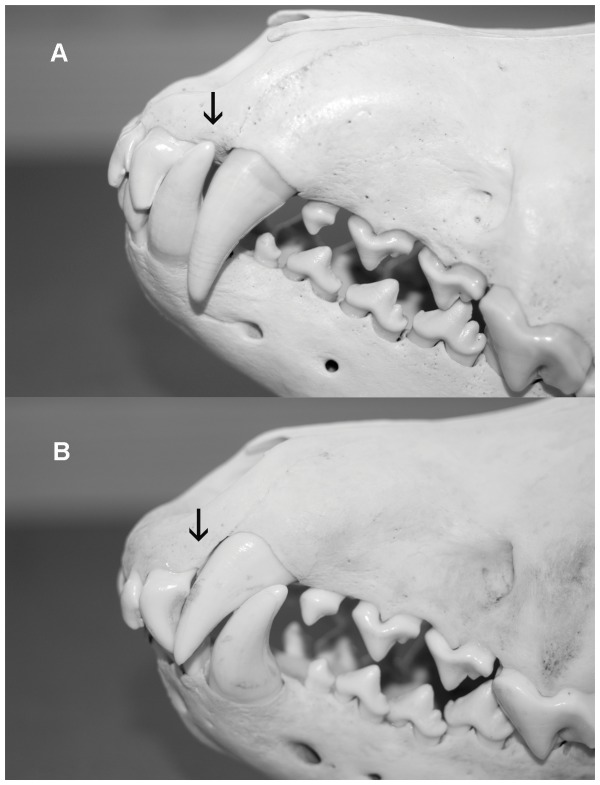
Normal canine occlusion in comparison with malocclusion of wolf NRM 20095298. The normal mandibular canine fits in the diastema between the maxillary third incisor and the maxillary canine, see arrow in panel (a). The malocclusion shows a mesioverted canine (tooth 204). Because of the anomaly the normal diastema is absent, see arrow in panel (b). This precludes normal mandibular canine occlusion that resulted in overshot jaw and palatal trauma from the right mandibular canine (tooth 404). The trauma led to periodontal damage of the right third maxillary incisor (tooth 103). Photos by J. Räikkönen.

**Figure 2:**
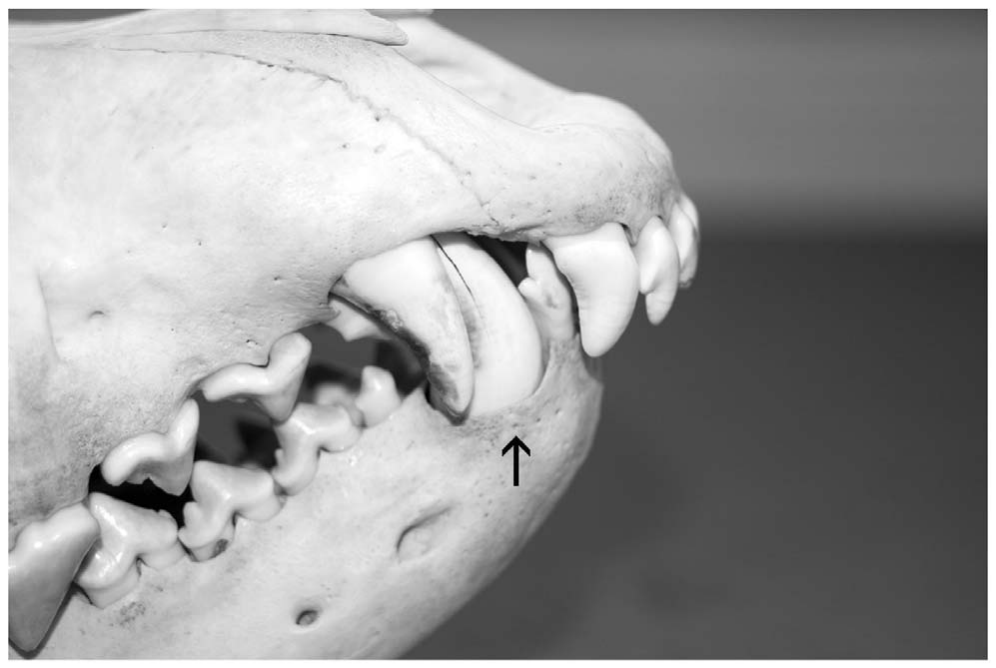
Wolf NRM 20115024 with pronounced overshot and an uncomfortable jaw closure. The mandibular canine (tooth 404), see arrow, caused palatal trauma near the maxillary canine (tooth 104). Photo by J. Räikkönen.

Three of the wolves with overshot exhibited mesioversion of one maxillary canine with resulting palatal injuries from the mandibular canines (Figs. 1 & 2). This type of palatal trauma can lead to several detrimental consequences [33].

Thirteen (8.9%) of the 146 wolves whose organs were necropsied at the Swedish National Veterinary Institute were characterized by some anomaly (including 2 with more than one anomaly). Of these 13 wolves, five had kidney defects (necropsy reports V49/02, V0661/07, V0377/08, V0295/09, V0141/11), two had heart defects (necropsy reports V49/02, V1349/06), and 8 exhibited cryptorchidism (necropsy reports V766/02, V2792/06, V0377/08, V2088/09, V0173/10, V0093/11, V0112/11, V4009/11). One of the wolves is known to have died from a heart defect (V49/02). Of the wolves exhibiting cryptorchidism, two were bilaterally cryptorchid and the remaining individuals were unilaterally affected. Bilateral cryptorchid animals are sterile and unilateral animals usually are fertile [34]. In addition to effects on fertility, cryptorchid individuals are at increased risk of developing associated pathology [34].

### Statistical analysis

We summarized the necropsy results by observing, for each wolf, its year of birth and the presence or absence (0, 1) of any kind of anomaly (vertebral, dental, or otherwise). We used these data and logistic regression to compare two hypotheses: (i) that the incidence of anomalies (*I_a_*) has been increasing over time, and (ii) that it has not been increasing. The predictor variable, year of birth, spanned a 32-year period (1978–2010). We fit two models to these data, one model assumes no temporal trend and includes only an intercept (*b_o_*), and the other model allows for the possibility of an increasing trend by also including a slope (*b_1_*). For both models, the predicted response is *I_a_*. We evaluated the relative merit of these two hypotheses using Akaike's Information Criterion, corrected for small sample size (AIC_c_) and Akaikeweights (*w*) [35]. Δ equals the AIC_c_ for the model of interest minus the smallest AIC_c_ for the set of models being considered. By definition, the best model has a Δ of zero, and models with Δ less than approximately 2 are generally considered worthy of consideration [36]. Akaike weights are useful because the ratio of *w_i_* to *w_j_* indicates how many times more likely model *i* is than model *j* [35].

For the model representing an increasing trend in *I_a_*, Δ is equal to 0, and the p-value for the slope is 0.05. For the model representing no trend in *I_a_*, Δ is equal to 4.3. The ratio of Akaike weights is 8.6, indicating that *I_a_* is 8.6 times more likely to have increased over time, than otherwise. The model indicating that *I_a_* had increased is not only the better of these two models, it is also reasonable to conclude that it is an adequate description of the temporal trend in *I_a_*. That is, the p-value for the Hosmer and Lemeshow test for goodness-of-fit is 0.15.

**Figure 3:**
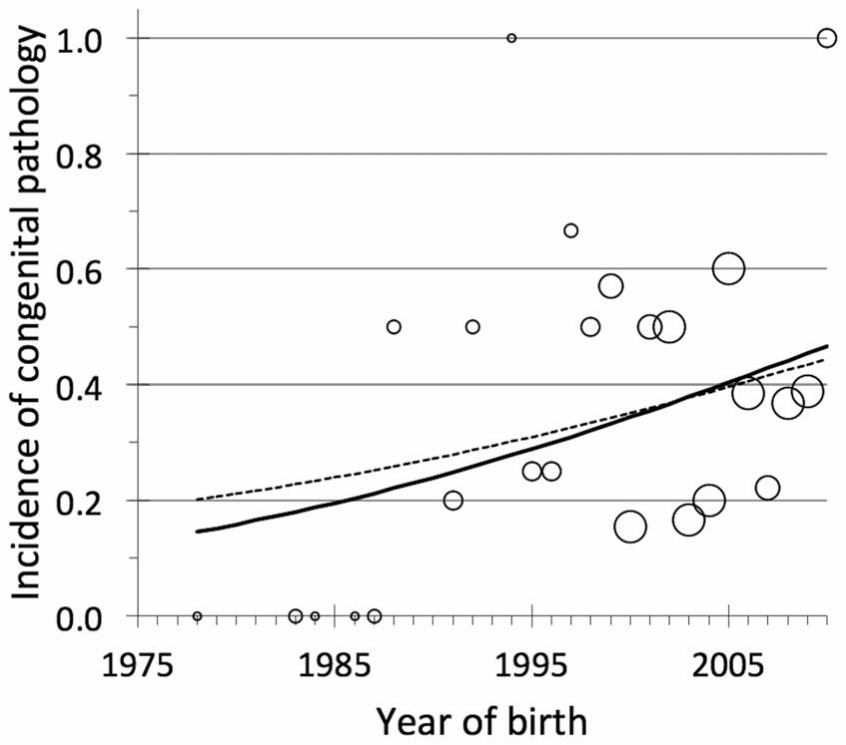
The relationship between year of birth and incidence of congenital pathology. We included 171 Scandinavian wolves born between 1978 and 2010. The wolf born in 1978 is the founding immigrant female. The solid line is the predicted logistic regression line, which includes an estimate for the slope and intercept. The dashed line is the weighted average of two models, the model that includes an estimate of the slope and intercept and a model including only an intercept (i.e., assumes no trend). Each circle is the proportion of wolves observed for a particular year with some kind of congenital pathology. The size of each circle represents the number of wolves observed each year. There are five sizes of circle, representing sample sizes of 1, 2–3, 4–6, 7–9, and 9 to 19.

To judge how much *I_a_* has changed over time, we first accounted for model uncertainty by considering predictions representing a weighted average of the two models, where the weights are *w* = 0.90 for the model *b_o_*, and *w* = 0.10 for the model without *b_o_* [35]. According to the weight-averaged model, *I_a_* tripled during the 32-year study period, from 13% in 1978 and it grew to 40% by 2010 (Fig. 3). If this trend continues for another two wolf generations (about 4 years per generation), the value of *I_a_* would be >50%. Each wolf generation (which is about 4 years), the odds (i.e., (*I_a_*/(1 – *I_a_*)) of being born with a congenital anomaly increases by 23%.

None of the three types of anomalies (dental, vertebral, soft tissue) was associated with sex (p-values were >0.31 for all three chi-squared test).

## Discussion

### Anomalies in Scandinavian wolves and examples from other populations

The occurrences of anomalies have been described in both outbred and inbred populations. A necropsy review of wolves (n = 241) and coyotes (*Canis latrans*) (n = 316) from outbred populations showed a very low frequency of congenital anomalies: one case of soft tissue (wolf) and one case of vertebral anomaly (coyote) was found [37]. However, the spinal columns and distal limb bones were not examined in detail unless there was some obvious abnormality [37]. The most prevalent type of vertebral anomaly in our study was LSTV, which is presumably hereditary in dogs (*Canis familiaris*) [30], [38]. The incidence of LSTV appears to be associated with the level of inbreeding for wolf populations [28]. Moreover, in the Scandinavian population the only numerical anomaly found is 6 lumbar vertebrae [this study] and the only LSTV type is sacralization (this study, [19]). In the Isle Royale population, the only numerical anomaly is 8 lumbar vertebrae and the only LSTV type is lumbarization [28]. An interesting observation to this is that different populations of purebred dogs seem to exhibit higher incidences of either sacralization or lumbarization [39].

Numerical variation of minor teeth with lesser importance (to survival), like premolars and small molars found in our study, have been reported fairly often in large outbred populations of wolves [40]–[43]. Three wolves exhibited mesioversion of canines. This anomaly, that can cause injury because of the resulting malocclusion, is prevalent in Shetland sheepdogs [44] and has to our knowledge never been described in wolves before.

A study of 500 wolf skulls from Russia and bordering countries [41] found at least 3.0% with some form of malocclusion. This may have been an underestimate, because it is unclear whether all types of malocclusions were reported in that study. A high incidence of malocclusions, 16.7% (n = 72), has been reported in an inbred population of red foxes (*Vulpes vulpes*) [45]. This frequency is in contrast to outbred foxes found with malocclusions, 0.1% (n = 785), studied by [46].

Among the reported soft tissue anomalies the cryptorchidism, i.e. retained testes, was the most prevalent. Cryptorchidism have been reported in several species and the prevalence seem to be <5% in most species and breeds [47]. But highly inbred populations have been found with high frequencies [48], [49]. Cryptorchidism is reported as a hereditary and clinical problem in dogs. [34]. It is suggested that cryptorchidism is a heritable condition but other suggested etiologies also exist like fetal exposure to endocrine disruptor agents [47]. Also other factors have been reported that may result in this anomaly (see [50] and references therein). This means it is possible that the individuals found in this study with cryptorchidism may have different etiologies.

There is good reason to believe the incidence of anomalies among Scandinavian wolves has been increasing substantially over the past three decades. Some of the anomalies we observed can have an impact on fitness e.g., heart defect, LSTV, cryptorchidism. These are anomalies that have been reported to be a problem in purebred dogs [51], [30], [34]. The fitness effects of other anomalies are less clear. Still other anomalies likely have no direct effect on fitness (e.g., minor tooth anomalies, thoracolumbar and sacrococcygeal transitional vertebrae), but may be an indication of high levels of inbreeding, much like the occurrence of anomalous coccygeal vertebrae in Florida panthers at the base of their tails [49].

Each kind of anomaly is also likely associated with a different genetic basis. This is important because inbreeding depression and founder effects on any particular trait for any particular population is the outcome of a highly stochastic process [52], [53]. In general, the negative effects of inbreeding can be difficult to detect, even when they are ecologically significant (e.g., [54]). For these reasons, the failure to detect the effect of these genetic processes at a single trait is not a reliable basis for concluding a population is unaffected. Consequently, our ability to detect the increasing trend in incidence of anomalies for the Scandinavian wolf population was likely facilitated by having considered multiple kinds of anomaly. A more precise understanding of inbreeding depression would be had by relating the incidence of anomalies to the inbreeding coefficient of each individual wolf in the sample. That analysis is beyond the scope of this paper, but would likely be worthwhile.

### Basis of conservation goals

Sweden's goal for wolf conservation is to limit wolf abundance to just over 200 wolves through the use of hunting. Hunting and limiting abundance are considered critical for controlling human intolerance of wolves, which is a serious threat to wolf conservation. Population viability analyses also indicate that limiting abundance at ∼200 wolves will result in a very high chance of wolves continuing to persist in southern Scandinavia [55]. However, the risk that genetic deterioration could compromise wolf vital rates, to the point of risking extinction, is also widely accepted [1], [2]. There is reason to think this risk can be mitigated through regular genetic rescue, facilitated by human-assisted migration (e.g., [14], [54]). Sweden's conservation goals seem appropriate if population health is more-or-less limited to ensuring presence of wolves in southern Sweden, i.e., merely avoiding extinction of remnant populations.

Population health may, however, entail more than merely avoiding extinction. Maintaining population health may also preclude actively limiting abundance to the point that genetic deterioration causes reduced vital rates [18] or results in a high incidence of abnormal phenotypes like those observed here (Fig. 3). In particular, to avoid further rise of the inbreeding rate the population must increase and a continuous gene flow must be secured [56]. If conservation is about restoring population health, and if population health precludes this kind of curtailment (limiting abundance), then Sweden's conservation goals seem inadequate.

This standard for conservation might be unrealistically high if, for example, it was impossible to secure enough habitat for a larger population. This is not the case for Scandinavian wolves. Similarly, if poaching limited abundance, and if managers were unable to reduce rates of poaching, then one might have to concede that population health is not achievable. Again, this is not the case. While poaching has affected the growth rate of wolves, it has not, so far, limited abundance [25].

Achieving conservation goals requires social tolerance. Critical levels of social tolerance can be lost, and a conservation backlash can occur, if conservation goals are set too high. A controversial strategy for avoiding conservation backlash is to look to social tolerance (sometimes called social carrying capacity) as a means for informing, or even determining, conservation goals [57], [58]. This seems to characterize the Swedish goals for wolf conservation; an approach analogous to reducing crime rates by legalizing illicit activities. Most certainly, conservation should entail socially-just plans for minimizing the gap between social carrying capacity and the requirements for population health. Social tolerance is not, however, a basis for understanding what constitutes population health or a basis for setting conservation goals, it is rather a basis for understanding the obstacles to achieving conservation goals.

Wolf conservation in Scandinavia and elsewhere raises many important issues, such as how should the precautionary principle be applied?, is it wrong to hunt wolves, in principle?, what should be the extent and method of lethal control in managing wolf-human conflicts?. Each issue deserves full attention. However, the concern to which we draw attention is not the means used to achieve conservation goals. Instead, we raise more basic concerns: should the goal for conserving populations be merely avoiding the risk of extinction, or should it be the maintenance and restoration of population health? If the more appropriate goal is the later, then it is important to understand what population health entails?

For example, population health would seem to require a population to maintain abundance without direct assistance from humans (e.g., regular additions from a captive population) to offset low reproduction or high mortality. The basis for such thinking is tied to the most basic and general equation in population biology [59]: *N_t_*_+1_ = *N_t_*+*B_t_*–*D_t_*+*I_t_*–*E_t_*, where *N* is abundance, and the remaining symbols are the number of births, deaths, immigrants, and emigrants. This equation highlights that reproduction and mortality are two of the three fundamental processes that comprise a population. By identical reasoning, population health would also seem to require a population to exhibit critical levels of dispersal on its own.

Requiring natural dispersal as a standard for conservation might be misplaced if something about the natural history of a population precluded such dispersal. But wolves are capable dispersers. Moreover, the main limitation on dispersal in Scandinavian wolves is anthropogenic mortality. The criteria for conservation success should be removal of the threat, not human-assisted migration. Abdicating that standard is analogous to believing that a population has been successfully conserved even if its viability depended on regular additions from a captive population.

### Achieving conservation goals and benchmarks

From the broadest perspective, Scandinavian wolves represent an important risk of conservation failure. The failure here is to distinguish conservation goals from what we might call conservation benchmarks. Conservation goals should be based on population health. In the particular case of Scandinavian wolves, population health includes the absence of inbreeding depression. Defining population health in general is difficult, but some important guidelines are that population health:

- It is more than mere persistence, for the same reason that an individual's health entails more than mere existence.
- It includes population viability, the 3Rs (representation, resiliency, and redundancy; [60]), genetic health, ecological functioning, and a healthy intact social structure.
- It is not defined by the complete absence of human impact, nor is it readily overridden by social or political interests.

A particularly difficult challenge is to know how much human impact a population can endure and remain healthy. In some cases, population health cannot be achieved in what is determined to be a socially-just manner or without infringing upon the vital or non-vital interests of some humans. Such cases do not, however, warrant the relaxation of conservation goals. Instead they warrant conceding the inability to achieve conservation, or conceding that population health is less important than the human interest with which it is in conflict. Such a dilemma represents a failure and a tragedy. It would be wrong to hide the failure by lowering the standards for conservation. Some conservation goals may be attainable, but not in the foreseeable future. In such cases, it is appropriate to develop benchmarks that are achievable in the foreseeable future as a way of achieving conservation, one step at a time.

With these principles in mind, human-assisted migration is the realization of an important conservation benchmark. Even if the benchmark has been realized, the conservation goal (i.e., a healthy wolf population) has not yet been attained. Sweden is currently in the process of revising abundance goals for wolves [61] and should develop a new benchmark that is attainable in the foreseeable future and closer to the conservation goal.

Finally, the high rate of congenital anomalies that Scandinavian wolves suffer, (Fig. 3), is a manifestation of poor population health that would be mitigated by larger population size and increased immigration, insomuch as they would mitigate the genetic deterioration that is almost certainly the cause of many of these anomalies. For this reason, instituting a public harvest of wolves designed to limit abundance at this time is almost certainly inconsistent with the conservation goal of a healthy wolf population, insomuch as limiting abundance would exacerbate genetic deterioration, at least until the time when rates of natural immigration are great enough to support the population's genetic health and the conservation status is favourable.
